# GPS-Supported Visual SLAM with a Rigorous Sensor Model for a Panoramic Camera in Outdoor Environments

**DOI:** 10.3390/s130100119

**Published:** 2012-12-21

**Authors:** Yun Shi, Shunping Ji, Zhongchao Shi, Yulin Duan, Ryosuke Shibasaki

**Affiliations:** 1 Center for Spatial Information Science (CSIS), University of Tokyo, Chiba 277-8568, Japan; E-Mails: shiyun@iis.u-tokyo.ac.jp (Y.S.); duan@iis.u-tokyo.ac.jp (Y.D.); shiba@iis.u-tokyo.ac.jp (R.S.); 2 School of Remote Sensing and Information Engineering, Wuhan University, Wuhan 430079, China; 3 Department of Environmental and Information Studies, Tokyo City University, Yokohama 222-0033, Japan; E-Mail: shizc@tcu.ac.jp

**Keywords:** panoramic camera, visual SLAM, bundle adjustment, GPS

## Abstract

Accurate localization of moving sensors is essential for many fields, such as robot navigation and urban mapping. In this paper, we present a framework for GPS-supported visual Simultaneous Localization and Mapping with Bundle Adjustment (BA-SLAM) using a rigorous sensor model in a panoramic camera. The rigorous model does not cause system errors, thus representing an improvement over the widely used ideal sensor model. The proposed SLAM does not require additional restrictions, such as loop closing, or additional sensors, such as expensive inertial measurement units. In this paper, the problems of the ideal sensor model for a panoramic camera are analysed, and a rigorous sensor model is established. GPS data are then introduced for global optimization and georeferencing. Using the rigorous sensor model with the geometric observation equations of BA, a GPS-supported BA-SLAM approach that combines ray observations and GPS observations is then established. Finally, our method is applied to a set of vehicle-borne panoramic images captured from a campus environment, and several ground control points (GCP) are used to check the localization accuracy. The results demonstrated that our method can reach an accuracy of several centimetres.

## 1. Introduction

Imagery from mono or stereo cameras has been the main data source for many applied science fields, such as robotics, computer vision and photogrammetry. Many research studies related to Simultaneous Localization And Mapping (SLAM) based on mono cameras [1,2] and stereo cameras [3,4] have been presented in recent decades. At the same time, multi-camera rigs (e.g., panoramic cameras) have increasingly been used for a wide range of research and applications because of their advantages, including omni-directional imaging, rotation invariance, and long baselines. However, the applications of SLAM with panoramic cameras should be studied theoretically because they use a different sensor model than mono/stereo cameras.

An ideal geometric sensor model of a panoramic camera has one projection centre, and all of the light beams satisfy co-linearity conditions or a pin-hole model and project the real world onto a spherical surface. This is a perspective transformation but is not projected onto a plane as in a mono/stereo camera. Geyer and Daniilidis give detailed projective geometry for a catadioptric sensor and emphasize the duality [5]. Another work given by Barreto and Araujo studies the geometry of the central catadioptric projection of lines and its use in calibration [6]. Spherical perspective transformation functions and homographies are also given by Mei *et al.* [7]. Kaess and Dellaert used a multi-camera rig (panoramic camera) for SLAM with an ideal spherical sensor model [8]. Paya *et al.* concentrated on the global description of each omni-directional image but still used the ideal sensor model [9]. Gutierrez *et al.* concentrate on the rotation and scale invariance of descriptor patches with a spherical camera model [10]. However, because of manufacturing constraints, it is almost impossible to guarantee that the projection centres of the individual lenses will be located at the same spherical centre. Thus, the rays will introduce a bias because the vertexes are moved from the mono-lens centre to the panoramic centre (additional explanations are provided in Section 2). In this paper, we will present the first general and rigorous sensor model for a panoramic camera to our knowledge.

Motion and structure estimations from a moving vehicle with a camera or several cameras have different applications in different research fields. In computer vision, this topic is called structure from motion (SFM); in robotics research, it is called SLAM (this term is used in this paper). Two common solutions to the SLAM problem are filtering and bundle adjustment (BA) [11]. When computation costs and real-time performance are considered, filtering is the more commonly used method of SLAM, and most studies utilise Kalman [12–14] or particle [15,16] filters. BA is more accurate and theoretically rigorous than filtering because filter-SLAM marginalises the previous information, and BA-SLAM keeps the global optimum [11,17], proves that all of the corresponding rays intersect correctly, and avoids matching and model errors. In recent years, several articles have studied BA-SLAM [17,18]; however, because of error propagation and the assumption of Gaussian distributions, a longer trajectory will generate more uncertainties in both filtering and BA. If a global optimum must be obtained with high precision, more constraints should be added to eliminate the accumulated uncertainties. Common constraints include closet, GPS, IMU, ground control points (GCP), landmarks and georeferenced maps.

Providing SLAM with global georeferencing information not only constrains the propagating uncertainties but also allows the extra spatial information to be used in more applications. Many researchers have studied SLAM with several types of geo-information, particularly maps and GPS. In [3], 2D road maps are used as the geographic reference for global vehicle localization with 3 degree of freedom (3DoF) particle filtering. Miller *et al.* [19] presented a similar map-aided approach for visual SLAM with particle filtering but combined it with GPS data. In [20], a method to recover position and attitude using a combination of monocular visual odometry and GPS measurements was presented, and the SLAM errors were carefully analysed after filtering. However, the egomotions were obtained with two-frame homography, which introduces both model error and matching errors and impacts the filtering results. In [21], two stages of filtering were used to improve the GPS location accuracy using an inertial navigation system and wheel encoders, and the SLAM solution was improved with a 3DoF model. Schleicher *et al.* [22] presented a real-time EKF hierarchical SLAM combined with GPS data, but the altitude information provided by the GPS were not used.

**Figure 1.**
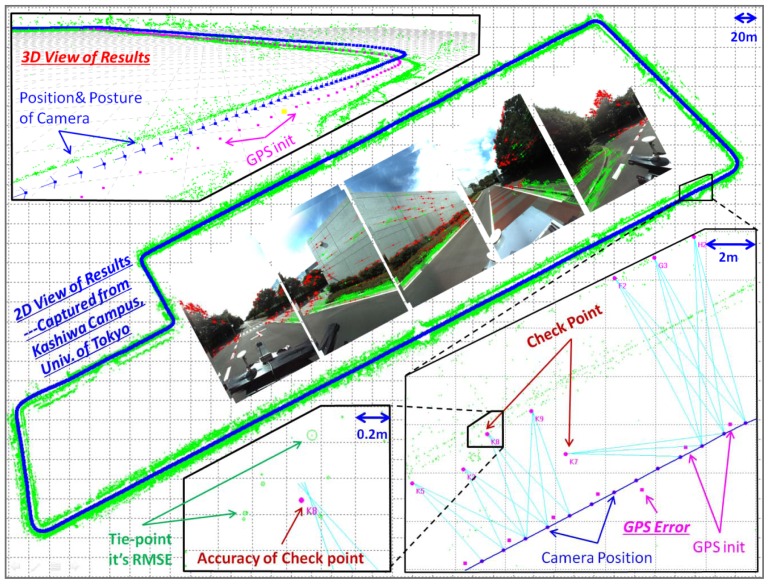
An overview of the results of our proposed method. The blue line in the middle represents the trajectory through the Kashiwa campus of the University of Tokyo, and the nearby green circles are the tie-points. The separate images in the route are from the 5 mono-lenses. Green dots indicate correctly matched tie-points with good distributions, and red dots indicate mismatched points that have been excluded from the error detection steps. A 3D view of the results is shown in the top left corner; blue dots represent the position and posture after SLAM, and pink dots represent the GPS route. The two boxes in the bottom right are the zoomed area in which the GCPs are included. The light green corresponding rays intersect correctly in the right box, and the RMSE of the tie-points and check points both reach an accuracy of several centimetres with a grid scale of 0.2 m (left box).

Although homography and the 3DoF models without altitude information used by these articles reduce the computation cost greatly, they all presume a planar Earth surface and may introduce large errors in elevation. Furthermore, these methods all use filtering methods. To our knowledge, no article has studied GPS-supported BA-SLAM. However, GPS-supported BA-SLAM should have a higher accuracy than filter-SLAM because of the theoretical rigor of BA itself. In this paper, we study a GPS-supported BA-SLAM method in which a 6DoF model is embedded, a rigorous sensor model is applied as the geometric projection model, and GPS data are combined with ray observations as additional restrictions for global optimisation and georeferencing. Finally, several ground control points (GCPs) are measured manually to check the absolute accuracy of the GPS-supported BA-SLAM method. Figure 1 shows the results of using our GPS-supported BA-SLAM on the Kashiwa campus of Tokyo University. The green circles represent the ground features/landmarks, and the radius shows the error, which is very small (average 1.6 cm). The thick blue line in the middle represents the road route, which is approximately 1.8 km long. The error of the check points has an accuracy of several centimetres.

The paper is structured as follows: Section 2 introduces a common dioptric panoramic camera and establishes a camera model that is more rigorous than the ideal model. Section 3 presents a stereo co-planarity (or epipolar constraint) that is more rigorous than the ideal co-planarity. Section 4 addresses the bundle algorithm supported by GPS, and Section 5 presents the results of the mapping and localization experiments. All of these experiments were carried out using a vehicle platform that consists of a multi-rig camera and GPS receiver. Finally, we present the conclusions and future work in Section 6.

## 2. Monocular Ideal Sensor Model *vs.* Rigorous Sensor Model of a Panoramic Camera

### 2.1. Projection from Fish-Eye Lenses to Panoramic Camera

**Figure 2.**
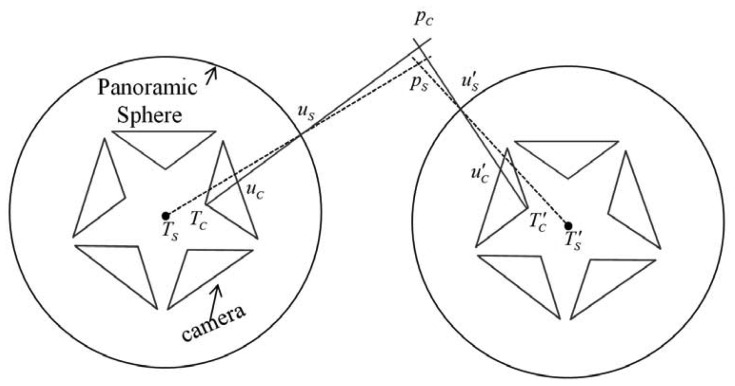
Representation of a panoramic camera consisting of five mono cameras. The dashed line on the left indicates an ideal ray corresponding to ideal sensor model that passes through the panoramic centre *T_S_*, a point on the panoramic sphere *u_s_* and the object *p_s_*. In reality, *u_s_* is imaged from the mono camera, and the projection centre is ***T****_c_*; the real ray is represented by the solid line corresponding to our rigorous sensor model and passes through ***T****_c_*, *u_s_* and *p_c_*. Two errors are introduced by the ideal model: one is the ray direction bias, and the other is the position offset of the landmarks.

As shown in Figure 2, the panoramic camera is composed of five separate fish-eye lenses. *T_S_* is the centre of the panoramic sphere. A two-step transformation is carried out to establish the relationship between fish-eye cameras and panoramic camera. In the first, the fish-eye image coordinates are transformed to the ideal plane camera coordinates, while the second transforms the plane coordinates to the uniform panoramic coordinates. Equation (1) describes how an image point *u_c_*with a coordinate vector ***u*** in a separate lens is projected to *u_s_* with a coordinate vector ***X*** = [*x*, *y*, *z*]^T^ in the panoramic sphere. ***K****_c_* is the transformation matrix from the image coordinate ***u*** in the fish-eye camera *c* to the corresponding undistorted plane coordinate and includes such parameters as radial distortion, tangential distortion and principal point offset [23]. ***R****_c_* and ***T****_c_* are the rotation matrix and translation vector from the coordinates of the ideal plane camera *c* to the panoramic coordinates, respectively. ***K****_c_*, ***R****_c_*, ***T****_c_* are fixed values because of the advanced calibration, and *k* is the scale factor from the ideal plane to the panoramic sphere coordinate, which varies with different points and can be calculated associated with Equation (2) which describes that ***X*** is on the panoramic sphere with a certain radius *R*. It should be mentioned that the panoramic coordinate ***X*** for a certain image point, is the same both in ideal and rigorous sensor models:

(1) $$X=kR_{c}K_{c}u+T_{c}$$

(2) $$x^{2}+y^{2}+z^{2}=R^{2}$$

### 2.2. Ideal Panoramic Sensor Model

The common ideal panoramic sensor model Equation (3) is the perspective transformation between an arbitrary 3D point *p_s_* with coordinate vector ***X****_A_* in the object space and the corresponding panoramic point *u_s_* with coordinate vector ***X*** obtained from Equation (1), which passes through the panoramic center *T_s_*. ***R*** and ***T*** are the rotation matrix and translation vector, respectively, and *λ* is the scale from the panoramic coordinate to object coordinate:

(3) $$\lambda X=R^{T}(X_{A}-T)$$

However, Figure 2 shows that two system errors occur. First, the rays are moved forcibly. The ray ***T****_c_u_s_*, which passes through the centre of the separate lens (shown by the solid line) is regarded as ***T****_s_u_s_* which passes through the panoramic centre (shown by the dashed line). This observation indicates that the ideal panoramic camera model is incorrectly constructed for the biased ray. The biased rays cause the second error that the real 3D point *p_c_* is translated to an incorrect position *p_s_*. However, the projection centres of the separate fish-eye cameras and the panoramic centre are very close, and the angle between ***T****_c_u_s_* and ***T****_s_u_s_* is very small, which may ensure that the system errors are limited to less than one pixel within a certain distance.

### 2.3. Rigid Panoramic Sensor Model

According to the analysis presented above, a rigorous sensor model should express the correct rays. The ray through ***T****_c_* and *u_c_* in a separate camera coordinate can be rigorous, but it loses the meaning of panoramic imaging. Thus, we construct the rigorous camera model under the uniform panoramic coordinate, which means that the co-linearity through ***T****_c_u_s_* is constructed:

(4a) $$T_{c}+\lambda (X-T_{c})=R^{T}(X_{A}-T)$$

(4b) $$\begin{array}{c} \frac{x^{\prime }-T_{x}}{z^{\prime }-T_{z}}=\frac{a_{11}(X_{A}-X_{S})+a_{21}(Y_{A}-Y_{S})+a_{31}(Z_{A}-Z_{S})-T_{x}}{a_{13}(X_{A}-X_{S})+a_{23}(Y_{A}-Y_{S})+a_{33}(Z_{A}-Z_{S})-T_{z}}\\ \frac{y^{\prime }-T_{x}}{z^{\prime }-T_{z}}=\frac{a_{12}(X_{A}-X_{S})+a_{22}(Y_{A}-Y_{S})+a_{32}(Z_{A}-Z_{S})-T_{y}}{a_{13}(X_{A}-X_{S})+a_{23}(Y_{A}-Y_{S})+a_{33}(Z_{A}-Z_{S})-T_{z}} \end{array}$$

In Equation 4(a,b), ***T****_c_* = [*T_x_*, *T_y_*, *T_z_*]^T^ is the translation vector between ***T****_c_* and ***T****_s_* and *X* represents the panoramic coordinate vector as in Equation (1). The vector *λ*(***X*** – ***T****_c_*) thus presents the true ray ***T****_c_u_s_* but in the mono camera coordinate system. The coordinate origin of the ray should be moved to the panoramic centre by adding a translation ***T****_c_*. Now ***X****_A_* represents the coordinates of the correct 3D point *p_c_*. The rigid perspective model under the panoramic coordinates is then constructed after rotation and translation with ***R*** and ***T***, respectively. Formulation (4b) is the algebraic form of (4a) in which the unknown *λ*is eliminated. Please note that the panoramic coordinate *X* obtained from Equation (1) should be consistent with ***T****_c_*, which is different from different mono-lenses.

In this paper, the rigorous sensor model (Equation 4(b)) will be used as the basic measurement equations for our GPS-supported BA-SLAM. For a BA method, the ray measurement equations (Equation 4(b)) are sufficient, and a motion model is not needed. However, BA requires initial values for the six unknown translation and rotation parameters. Rigorous co-planarity conditions will be introduced below to supply the initial values. The idea is similar to [24], in which epipolar constraints are used for stable estimation of camera trajectory.

## 3. Ideal Co-Planarity *vs.* Rigorous Co-Planarity of a Panoramic Camera

### 3.1. Ideal Panoramic Co-Planarity

Co-planarity, also called epipolar constraints, is a well-known geometric relationship between stereo-image pairs that reflects the two camera positions and the corresponding image coordinates in one plane. As described above, extra velocity and angular velocity are not needed as parameters of a motion model because a filter framework is not used and BA only needs the initial position and orientation vectors as inputs. The co-planarity will supply sufficient parameters for the image association and the initial values for BA.

Figure 2 shows two stereo panoramic images with a baseline *T_s_* $T_{s}^{\prime }$. We write ***B*** = [*B_X_ B_Y_ B_Z_*] and the corresponding rays ***T****_s_u_s_* as ***V***_1_ = [*X*_1_ *Y*_1_ *Z*_1_] and $T_{s}^{\prime }u_{s}^{\prime }$ as ***V***_2_ = [*X*_2_ *Y*_2_ *Z*_2_]. The vectors ***B***, ***V***_1_ and ***V***_2_ satisfy the epipolar constraints as follows:

(5) $$B•(V_{1}\times V_{2})=0$$

In Equation (5), $\left[\begin{array}{c} X_{1}\\ Y_{1}\\ Z_{1} \end{array}\right]=\left[\begin{array}{c} x_{1}\\ y_{1}\\ z_{1} \end{array}\right],\left[\begin{array}{c} X_{2}\\ Y_{2}\\ Z_{2} \end{array}\right]=R\left[\begin{array}{c} x_{2}\\ y_{2}\\ z_{2} \end{array}\right],\left[\begin{array}{c} x_{1}\\ y_{1}\\ z_{1} \end{array}\right],\left[\begin{array}{c} x_{2}\\ y_{2}\\ z_{2} \end{array}\right]$ are the coordinates of the corresponding points and ***R*** is the rotation matrix between the two images.

If Equation (5) is expanded by the third line of the determinant, Equation (6) can be deduced, in which *a*, *b* and *c* are determined by the values of ***V***_1_ and ***R***. Equation (6) represents a 3D plane that passes through the coordinate origin. Combined with Equation (2), the panoramic sphere equation, we conclude that the epipolar line of ideal panoramic stereo images is a large circle through the projection's centre. Equation (6) can be used as a geometric constraint for image matching and outlier elimination:

(6) $$ax_{2}+by_{2}+cz_{2}=0$$

### 3.2. Rigorous Panoramic Co-Planarity

We can see that Equation (5) is not rigorous because the actual rays do not pass through the centres *T_s_*, $T_{s}^{\prime }$ of the panoramic spheres but rather through the projection centres of the separate lenses *T_c_*, $T_{c}^{\prime }$. Thus, the vectors ***B***, ***V***_1_ and ***V***_2_ all have errors. Because the monocular rigorous camera model is constructed in uniform panoramic coordinates, we construct the co-planarity in the same coordinates.

First, the actual corresponding rays ***V***_1_ and ***V***_2_ should pass through the projection centres of the separate cameras as in Equations (7) and (8). In addition, B should be the baseline between the separate lenses but be in the uniform panoramic coordinates, as in Equation (9):

(7) $$V_{1}=X_{1}-t_{1}=\left[\begin{array}{c} x_{1}-T_{x_{1}}\\ y_{1}-T_{y_{1}}\\ z_{1}-T_{z_{1}} \end{array}\right];$$

(8) $$V_{2}=R(X_{2}-t_{2})=R\left[\begin{array}{c} x_{2}-T_{x_{2}}\\ y_{2}-T_{y_{2}}\\ z_{2}-T_{z_{2}} \end{array}\right]$$

(9) $$B=S_{1}-S_{2}=\left[\begin{array}{c} BX+{T^{\prime }}_{x_{2}}-T_{x_{1}}\\ BY+{T^{\prime }}_{y_{2}}-T_{y_{1}}\\ BZ+{T^{\prime }}_{z_{2}}-T_{z_{1}} \end{array}\right]$$

In Equations (7–9):

$$S_{1}=T_{1}+t_{1}=\left[\begin{array}{c} X_{S_{1}}+T_{x_{1}}\\ Y_{S_{1}}+T_{y_{1}}\\ Z_{S_{1}}+T_{z_{1}} \end{array}\right];S_{2}=T_{2}+t_{2}^{\prime }=\left[\begin{array}{c} X_{S_{2}}+{T^{\prime }}_{x_{2}}\\ Y_{S_{2}}+{T^{\prime }}_{y_{2}}\\ Z_{S_{2}}+{T^{\prime }}_{z_{2}} \end{array}\right];t_{2}^{\prime }=\left[\begin{array}{c} {T^{\prime }}_{x_{2}}\\ {T^{\prime }}_{y_{2}}\\ {T^{\prime }}_{z_{2}} \end{array}\right]=\text{R}\left[\begin{array}{c} T_{x_{2}}\\ T_{y_{2}}\\ T_{z_{2}} \end{array}\right]$$

$\left[\begin{array}{c} X_{S_{1}}\\ Y_{S_{1}}\\ Z_{S_{1}} \end{array}\right],\left[\begin{array}{c} T_{x_{1}}\\ T_{y_{1}}\\ T_{z_{1}} \end{array}\right],\left[\begin{array}{c} X_{S_{2}}\\ Y_{S_{2}}\\ Z_{S_{2}} \end{array}\right],\left[\begin{array}{c} T_{x_{2}}\\ T_{y_{2}}\\ T_{z_{2}} \end{array}\right]$ are the panoramic projection centres and offsets of two stereo images from the mono-lens to the panoramic camera, respectively.

If the vectors ***B***, ***V***_1_ and ***V***_2_ are calculated as Equations (7–9), Equation (5) will be a rigorous model for stereo panoramic co-planarity. We can also calculate the epipolar line by expanding Equation (5):

(10) $$ax_{2}+by_{2}+cz_{2}=d$$

In Equation (10), the constant term *d* is determined by ***R***, and the offsets between the panoramic centre and the projection centres of the separate cameras do not equal zero. Thus, the epipolar line is not a large circle around the panoramic sphere. However, *d* is typically a very small value, which makes the epipolar very similar to a large circle.

In this paper, the rigorous panoramic co-planarity Equations (7–9) is used to calculate the relative translation ***B*** and the orientation ***R*** between stereo images and as a geometric constraint to eliminate outliers.

## 4. GPS-Supported Visual SLAM with the Rigorous Camera Model

**Figure 3.**
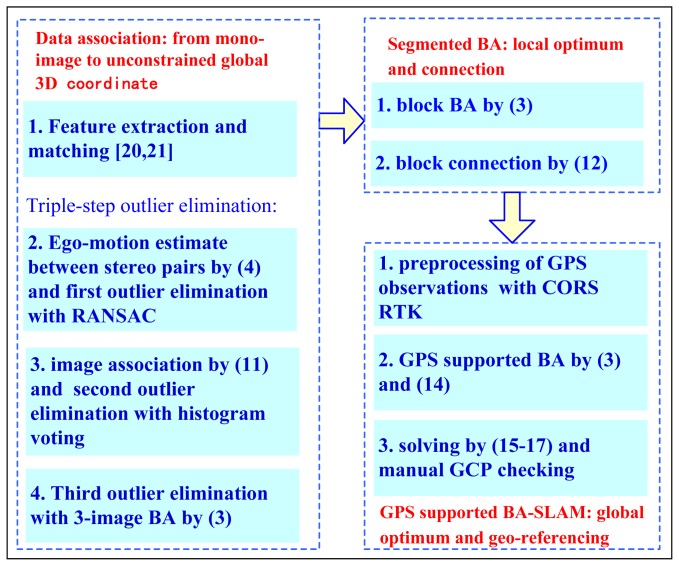
The main workflow of GPS-supported BA-SLAM.

This paper focuses on accurate global localization in large-scale outdoor environments using GPS-supported vehicle-borne panoramic imagery. The GPS-supported BA method has been used for aerial triangulation for many years, but to date, it has not appeared in the field of SLAM research to our knowledge. Filtering has been the only method used to combine these two observations. In this paper, we combine GPS data with image observations in a BA framework, and three carefully designed steps, accurate data association, segmented BA and GPS-supported BA are used to form an integral workflow (Figure 3).

### 4.1. Data Association

Data association is a key point in SLAM. The data should be verified so that all mismatched features are eliminated correctly and so enough information remains. We introduce a three-step outlier elimination process to ensure that all of the matched features are correct.

The data association begins with feature extraction and matching with GPU-SIFT [25,26]. The RANSAC method [27], which is embedded with geometric constraints Equation (5), is used first for outlier elimination in each stereo model, and then, the relative motion estimate (6DoF) is obtained. Secondly, the corresponding 3D points in the adjacent stereo models are used to calculate the unknown scale between stereo models, and only one point is needed for solving the scale [24]. However, there may be rays that cannot satisfy the multi-intersection. Since redundant observations are provided by many 3D points, a histogram voting method is introduced for error elimination, and per 3D point votes once for a certain scale, which is similar to [28] but solves for the optimal scale. After the relative 6DoF are regularised to a uniform scale, all of the images are then associated to the coordinates of the first image according to Equation (11):

(11) $$\begin{array}{l} \left[R_{i}|T_{i}]=[R_{i-1,i}R_{i-1}|T_{i-1}+R_{i-1,i}T_{i-1,i}\right]\\ \left[R_{0}|T_{0}]=[E|O\right] \end{array}$$

In Equation (11), ***R****_i_*,***T****_i_* represent the rotation and translation of the *i*-th image to the global coordinates, respectively, and ***R****_0_*,***T****_0_* represent the first image.

**Figure 4.**
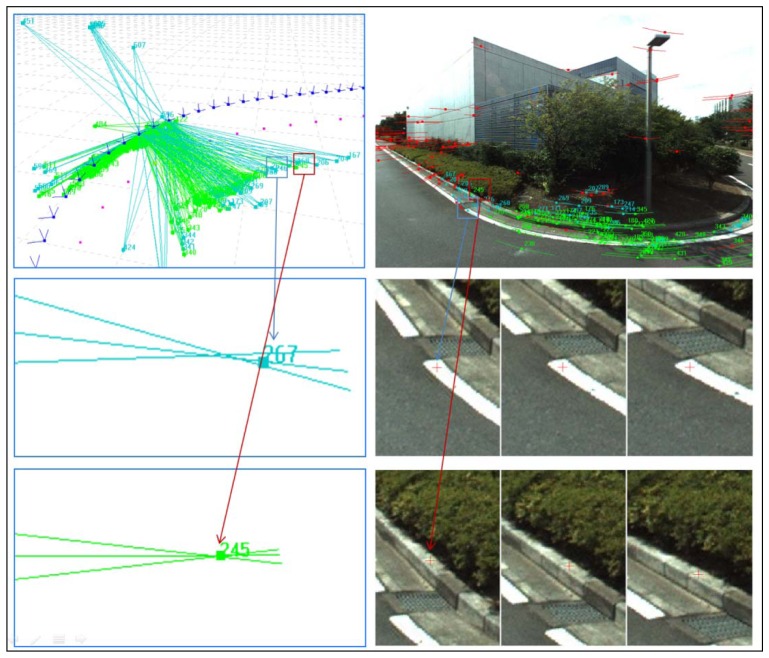
A 3D view of successfully matched tie-points (green). The points excluded by RANSAC (the first outlier elimination step) and histogram voting (the second step) are shown in red, and those excluded by BA (the third step) are shown in blue. The blue rays represent features that cannot intersect precisely, such as feature 267. Feature 267 may be regarded as correctly matched (right-middle images), but the lack of information about features in the window may introduce a bias of one or more pixels, which causes a slight intersection error (left-middle image). In contrast, feature 245 has a better texture and intersects precisely.

After the outlier elimination in the first two steps, the large errors are all removed correctly; these errors are shown as red points in Figure 4. However, there are still some small errors that will impact the accuracy of the next processes, which are shown as blue points in Figure 4. The error elimination is carried out a third time to eliminate these points. We only execute rigid BA for 3 images according to Equation (4b) to ensure that all of the false correspondences are eliminated when the 3 rays do not intersect precisely. For example, tie-points with intersection errors greater than 0.03 m will be removed.

Figure 4 shows the results of the three-step outlier elimination method. The green points are those remaining after correct intersection of three rays. The red points are those excluded by the first two outlier elimination steps, and the blue points are those excluded by the third step. Figure 4 shows that the third step can eliminate slight errors caused by matching accuracy because of a lack of texture.

### 4.2. Segmented BA-SLAM

The biggest problem with a large-scale BA for SLAM is the accumulation of position and orientation uncertainties because of error propagation, which will prevent the iteration from converging because BA requires accurate initial values. In contrast, filtering methods, extended Kalman filtering and particle filtering always give a possible solution.

As described in several articles as [29,30], the segmentation method is used to divide the entire strip into several blocks for rapid convergence. For example, 100 images are examined as a block, BA is carried out using Equation (4b) and a local optimum is obtained for each block. The adjacent blocks are then connected as a whole. For example, the translation and rotation vectors of the second block will be transformed to the first block according to:

(12) $$[R_{i}|T_{i}]=[\Delta R_{1}R_{i}|\lambda_{1}\Delta R_{1}(\Delta T_{1}+T_{i})],i\in \text{BLOCK}\;2$$

In Equation (12), *λ*_1_, Δ***R***_1_, Δ***T***_1_ represent the difference of the scale, rotation and translation parameters between the two blocks, which can be calculated as a well-known 3D transformation according to the corresponding landmarks in two blocks:

(13) $$X_{2}=\lambda_{1}\Delta R_{1}(\Delta T_{1}+X_{1})$$

In Equation (13), ***X***_1_, ***X***_2_represent the corresponding landmarks from the first and second blocks, respectively, which were obtained by multi-intersection. A larger dataset of ***X***_1_, ***X***_2_ will provide a more robust solution, and we set the adjacent blocks with five overlapping images. After all of the blocks have been connected, a global BA result of the local optima can be obtained. Similar to global BA, segmented BA cannot reduce the accumulation of uncertainties. As in Equation (13), the errors of ***X***_1_ will be propagated to ***X***_2_ according to the error propagation law.

### 4.3. GPS-Supported BA-SLAM

After the segmented BA-SLAM, GPS will be introduced to obtain georeferencing information and reduce the accumulated uncertainties. The 6DoF of all the images are then translated to global coordinates with a polynomial interpolation according to GPS values, and looked as the initial values for GPS-supported BA. The GPS observations are preprocessed with CORS (Continuously Operating Reference Station)-supported [31] RTK [32] technology and can reach an accuracy of up to 0.1 m in good conditions. Generally, with one GPS receiver mounted on a vehicle, the GPS observation equations associated with 6DoF of a camera can be constructed as:

(14) $$\left[\begin{array}{l} X_{G}\\ Y_{G}\\ Z_{G} \end{array}\right]=\left[\begin{array}{l} X_{S}\\ Y_{S}\\ Z_{S} \end{array}\right]+R\left[\begin{array}{l} U\\ V\\ W \end{array}\right]$$

In Equation (14), *X_G_*, *Y_G_*, *Z_G_* and *X_S_*, *Y_S_*, *Z_S_* are the GPS observations and camera positions at each exposure time, respectively. ***R*** is the rotation matrix, and *U*, *V*, *W* represents the translation between the camera projection centre and the antenna centre of GPS receiver, which can regarded as fixed values because of the calibration that was performed in advance. When combined with (4b), the GPS-supported BA with the rigorous sensor model is obtained.

Because Equation (14) does not introduce new unknown parameters, Equations (4b) and (14) can be solved as a classic non-linear least-squares Gauss–Newton solution. The linear in Equation (15) are obtained after linearization with a Taylor-series expansion, in which ***x*** represents the unknowns of the features and ***t*** represents the six translation and rotation parameters. ***A*** and ***B*** are Jacobians of Equation (4b), ***L*** represents the constant terms, ***C*** is the Jacobian of Equation (14) and ***L****_G_* represents the corresponding constants. ***P*** and ***P****_G_* are the inverse matrices of the covariance matrix that describe the uncertainties of the ray observations and GPS observations, respectively. The normal equations are then constructed as Equation (16). Equation (16) contains two types of unknowns; typically, the unknown ***x*** is eliminated, and only ***t*** remains, as shown in Equation (17). After Equation (17) is solved with a sparse Cholesky solver as in [33], ***t*** is substituted into Equation (16) to solve for ***x***. It is time consuming to obtain an exact solution for ***P*** for every observation, particularly at a large scale. ***P*** is typically set to an identity matrix under the assumption that all observation errors are Gaussian and independently distributed. ***P****_G_* will be deduced according to the accuracy of the GPS against the accuracy of the ray observations. In our test, ***P****_G_* is between 0.1 and 1:

(15) $$\begin{array}{l} At+Bx=l,P\\ Ct=L_{G},P_{G} \end{array}$$

(16) $$\left[\begin{array}{cc} A^{T}PA+C^{T}P_{G}C & A^{T}PB\\ B^{T}PA & B^{T}PB \end{array}\right]\;\left[\begin{array}{c} t\\ x \end{array}\right]=\left[\begin{array}{c} A^{T}\text{PL}+C^{T}P_{G}L_{G}\\ B^{T}PL \end{array}\right]$$

(17) $$(A^{T}\text{PA}+C^{T}P_{G}C-A^{T}\text{PB}{\left(B^{T}PB\right)}^{T}B^{T}PA)t=A^{T}\text{PL}+C^{T}P_{G}L_{G}-A^{T}PB{\left(B^{T}PB\right)}^{T}B^{T}PL$$

## 5. Experiments

### 5.1. Test Design

**Figure 5.**
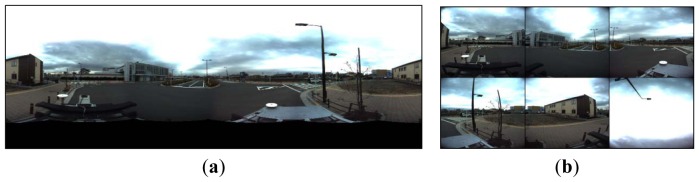
Panoramic image and separate images captured by the Ladybug system. (**a**) Panoramic image. (**b**) Images from 6 separate fish-eye lenses. The image aimed at the sky is not used in our SLAM.

**Figure 6.**
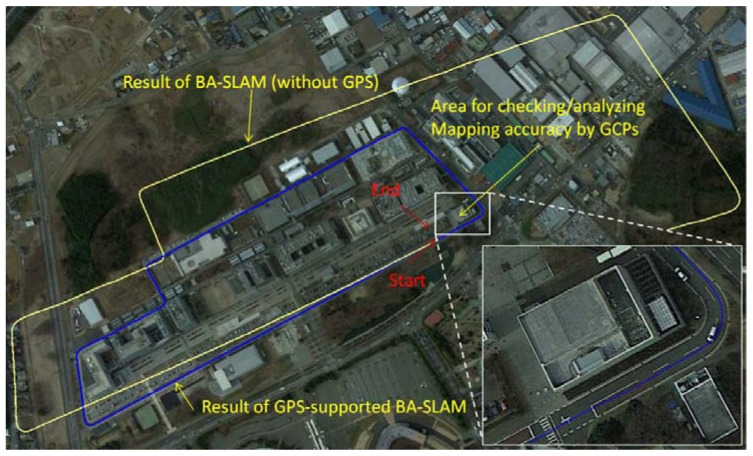
Results of the segmented BA-SLAM and GPS-supported BA-SLAM methods. The yellow line is the trajectory of the unconstrained results after data association and block BA. The start point is located in the correct position, but the trajectory shows a large accumulation of uncertainty in angle and scale. The blue line represents the trajectory after the GPS-supported BA-SLAM method is applied and shows a high level of accuracy. All eight GCPs are located in the enlarged area and are shown in Figure 7.

To test the proposed rigorous sensor model and its application in GPS-supported SLAM on a vehicle platform, we use PGR's Ladybug system [34], which consists of a multi-camera rig for panoramic imaging, as shown in Figure 5. The six separate fisheye images have a size of 1,616 pixels × 1,232 pixels, a 0.009 mm pixel resolution and 24-bit RGB colour resolution. The images that are aimed at the sky are not used. The focal length of the fisheye images is 3.3 mm, and the optimum radius of the panoramic sphere is 24 m. A dual-frequency GPS receiver is mounted on top of the car, and the distance between the GPS antenna centre and the camera centre is calibrated precisely in advance. The trajectory is shown as the blue line in Figure 6 from an overhead view in Google Earth. Adjacent images are taken at 1 m intervals over a total course length of approximately 1.8 km. For an off-line SLAM, the GPS observations are pre-processed using CORS RTK technology.

### 5.2. BA Results without GPS

After the three rounds of outlier elimination, all of the remaining corresponding rays intersect correctly, as shown in Figure 4. The red features in the images are regarded as outliers and are eliminated by RANSAC and histogram voting, and the blue points are eliminated by the 3-image BA because they exceed an intersection error of 0.03 m. The green points are the points remaining. The remaining features are located on both sides of the road and are seldom in front of or behind the car because features on the side of the road have larger intersection angles, which lead to higher intersection accuracy. In contrast, a small intersection angle causes a large uncertainty. Some of the red points in Figure 4 may have been correctly matched but were excluded only because they did not meet the accuracy threshold. The yellow trajectory in Figure 6 shows the results after data association and local BA and shows that the angle and scale uncertainties gradually accumulate, even if accurate ray observations are generated by the three-step outlier elimination process and local BA.

### 5.3. BA Results with GPS

The blue line in Figure 6 represents the trajectory after the GPS-supported SLAM is applied and shows a higher level of accuracy than the results of the segmented BA-SLAM. The quantitative results are shown in Table 1. The check errors of the GCPs are all less than 10 cm, with an average of 6.7 cm, which is similar in precision to measurements made with a total station system. This accuracy is sufficient for most applications.

**Figure 8.**
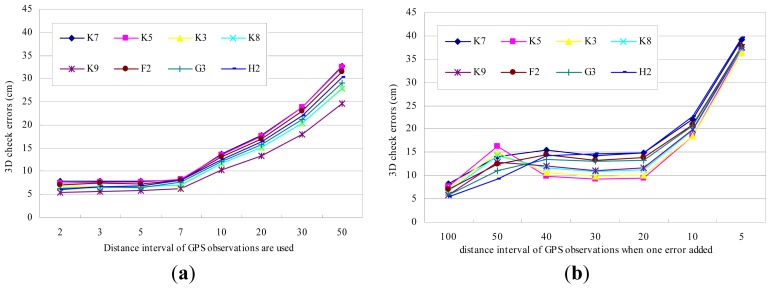
(**a**) Check errors *vs.* the number of GPS observations used. “Distance interval *n*” on the *X* axis means that one GPS observation is selected for every *n* m. The check errors of all 8 GCPs increase when the number of GPS observations is reduced but are still less than 0.35 m. (**b**) Check errors *vs.* number of gross errors added to the GPS observations. On the *X* axis, “Distance interval *n*” means that the gross error is added to one GPS observation every *n* meters. The check errors of all 8 GCPs increase when more GPS observations are given gross errors but are still less than 0.4 m.

**Figure 9.**
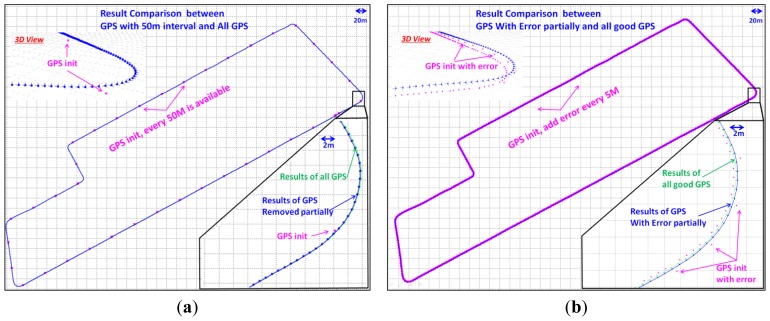
(**a**) Result comparison between GPS with 50 m interval and All GPS. In fact there are two trajectories with different colours, blue and green, which cannot be distinguished in a scale of 20 m. While at the zoomed area with a scale of 2 m, we can see the very slight difference. (**b**) Results comparison between GPS with errors introduced per 5 m and all good GPS. The same to (a), we can only distinguish the difference of trajectories in the zoomed area.

Because it is the only georeferencing information, the quality of the GPS observations is very important for the convergence of the union Equations (15) and the final localization accuracy. The effects of GPS on BA-SLAM should be carefully evaluated if too few GPS observations are obtained or there is insufficient accuracy due to multipath effects. We designed two tests to evaluate the GPS impact of our method. First, we gradually reduced the number of GPS observations and evaluated the localization results with different numbers of observations. As shown in Figure 8(a), eight assumptions were tested. For example, a “distance interval 2” on the *X* axis means that we only use one GPS observation for every two images/meters (one image is captured per meter), and “interval 50” means that one GPS observation is used every 50 meters. Figure 8(a) shows that the 3D check errors gradually increase with an increasing in the size of the interval. If the distance interval is less than 10, an accuracy of greater than 0.1 m can be reached; this level of accuracy is similar to that obtained when the results of all of the GPS observations are used (Table 1). With an interval of 50 m, the accuracy is approximately 0.3 m, which is sufficiently high for many applications; in this case, more than 30 GPS observations are enough for the large outdoor SLAM. Figure 9(a) is the comparison of the SLAM results between all GPS observations and observations with an interval of 50 m are used. The slight difference only can be distinguished at the zoomed area, which indicates good SLAM results of the whole trajectory even if GPS are sparsely resampled.

The second test examines the robustness of our method when gross errors/outliers exist in the GPS data. A gross error of 1 m (10 times the original GPS deviations) is introduced to selected GPS observations by rules, as shown in Figure 8(b). On the *X* axis, “distance interval 100” means that the gross errors are added to one GPS observation every 100 m, while “interval 5” means that one fifth of the observations are given gross errors. Figure 8(b) shows that the check errors gradually increase with a decrease in the size of the interval and reach an accuracy of approximately 0.37 m. These results show that even when the GPS observations contain many errors, our method can still provide acceptable localization results. Figure 9(b) is the comparison between GPS with errors introduced per 5 m and all good GPS are used. As in Figure 9(a), the slight difference can only be distinguished at the zoomed area, which proved the robustness of our GPS-supported BA method against gross errors.

## 6. Conclusions and Future Work

In this paper, we present a framework for GPS-supported BA-SLAM with a new rigorous sensor model for a panoramic camera. The test results show that our method is capable of obtaining global localization accuracy of several centimetres when GPS observations are favourable and demonstrate that our rigorous sensor model is both correct and effective. The tests show that our method is robust and provides an acceptable accuracy of several decimetres, even when GPS observations are partially unavailable or with big errors. The main contribution of this paper is that it is the first time that a GPS-supported BA has been used in a vehicle-based outdoor SLAM with a panoramic camera. This system may complement mainstream filtering solutions. The second contribution is that the paper proposes a new sensor model for panoramic cameras that is theoretically rigorous and considers the small offsets between the panoramic centre and the centres of the separate lens. The model may avoid slight but unnecessary system errors compared to the ideal sensor model.

Solutions based on BA may be more accurate than those using filters, but BA still has some shortcomings. BA requires accurate initial values to guarantee the convergence of the iteration. In our method, a three-step outlier elimination process is performed to guarantee that all of the tie-points are correct. Segmented BA has no trouble with good ray observations; in the global GPS-supported BA, however, Equation (15) depends on the consistency of the two observations, the rays and the GPS data. In Section 4, we verified that our method is robust regardless of a lack of GPS observations or if gross errors are introduced. However, the method will not generate satisfactory results if the GPS data contain excessive noise or conflict with the ray observations. In this case, BA-SLAM cannot provide results, and filter-SLAM is preferred because it can give a possible solution, though it may be unreliable.

Future work will focus on SLAM accuracy. Two problems must be addressed. First, the robust data association will be tested and improved in complicated environments, such as in a busy highway, where the large number of moving vehicles will be the greatest challenge. Second, tall buildings in cities may cause the GPS signals to be locked out for long periods. We will develop a reliable method to maintain the consistency of the local SLAM results with GPS and detect gross errors in the GPS observations automatically.

## Figures and Tables

**Figure 7.**
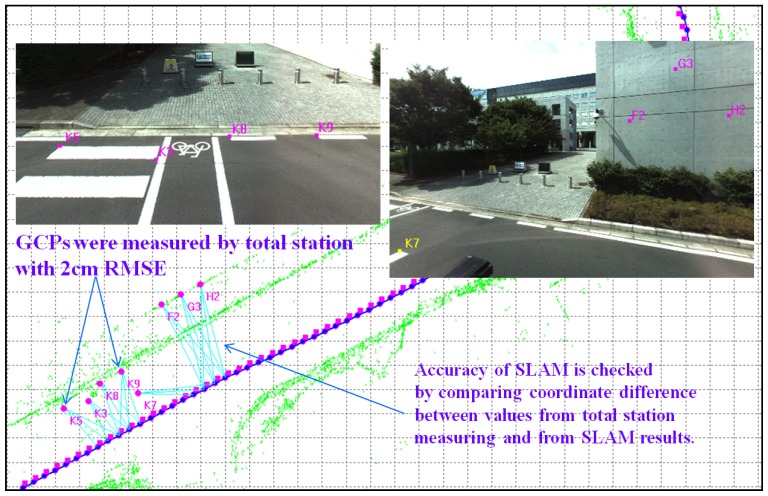
The eight GCPs, with accuracy up to 2 cm, are used in the experiments to check the accuracy of the GPS-supported BA-SLAM.

**Table 1.** Check errors of the eight GCPs.

**ID**	***D****_X_* **(cm)**	***D****_Y_* **(cm)**	***D****_Z_* **(cm)**	***D****_XYZ_* **(cm)**
K7	3.6	6.6	3.1	8.1
K5	3.8	6.0	1.7	7.3
K3	4.0	4.6	3.5	7.0
K8	4.4	4.2	2.3	6.5
K9	3.3	4.2	1.8	5.6
F2	−1.7	6.9	0.5	7.1
G3	−1.2	6.0	0.2	6.1
H2	2.4	5.2	−0.6	5.7

Average	3.0	5.5	1.7	6.7
